# Freezing of gait assessment with inertial measurement units and deep learning: effect of tasks, medication states, and stops

**DOI:** 10.1186/s12984-024-01320-1

**Published:** 2024-02-13

**Authors:** Po-Kai Yang, Benjamin Filtjens, Pieter Ginis, Maaike Goris, Alice Nieuwboer, Moran Gilat, Peter Slaets, Bart Vanrumste

**Affiliations:** 1 eMedia Research Lab/STADIUS, Department of Electrical Engineering (ESAT), KU Leuven, Andreas Vesaliusstraat 13, 3000 Leuven, Belgium; 2 Intelligent Mobile Platforms Research Group, Department of Mechanical Engineering, KU Leuven, Andreas Vesaliusstraat 13, 3000 Leuven, Belgium; 3 Research Group for Neurorehabilitation (eNRGy), Department of Rehabilitation Sciences, KU Leuven, Tervuursevest 101, 3001 Heverlee, Belgium

**Keywords:** Temporal convolutional neural networks, Freezing of gait, Parkinson’s disease, MS-TCN

## Abstract

**Background:**

Freezing of gait (FOG) is an episodic and highly disabling symptom of Parkinson’s Disease (PD). Traditionally, FOG assessment relies on time-consuming visual inspection of camera footage. Therefore, previous studies have proposed portable and automated solutions to annotate FOG. However, automated FOG assessment is challenging due to gait variability caused by medication effects and varying FOG-provoking tasks. Moreover, whether automated approaches can differentiate FOG from typical everyday movements, such as volitional stops, remains to be determined. To address these questions, we evaluated an automated FOG assessment model with deep learning (DL) based on inertial measurement units (IMUs). We assessed its performance trained on all standardized FOG-provoking tasks and medication states, as well as on specific tasks and medication states. Furthermore, we examined the effect of adding stopping periods on FOG detection performance.

**Methods:**

Twelve PD patients with self-reported FOG (mean age 69.33 ± 6.02 years) completed a FOG-provoking protocol, including timed-up-and-go and 360-degree turning-in-place tasks in On/Off dopaminergic medication states with/without volitional stopping. IMUs were attached to the pelvis and both sides of the tibia and talus. A temporal convolutional network (TCN) was used to detect FOG episodes. FOG severity was quantified by the percentage of time frozen (%TF) and the number of freezing episodes (#FOG). The agreement between the model-generated outcomes and the gold standard experts’ video annotation was assessed by the intra-class correlation coefficient (ICC).

**Results:**

For FOG assessment in trials without stopping, the agreement of our model was strong (ICC (%TF) = 0.92 [0.68, 0.98]; ICC(#FOG) = 0.95 [0.72, 0.99]). Models trained on a specific FOG-provoking task could not generalize to unseen tasks, while models trained on a specific medication state could generalize to unseen states. For assessment in trials with stopping, the agreement of our model was moderately strong (ICC (%TF) = 0.95 [0.73, 0.99]; ICC (#FOG) = 0.79 [0.46, 0.94]), but only when stopping was included in the training data.

**Conclusion:**

A TCN trained on IMU signals allows valid FOG assessment in trials with/without stops containing different medication states and FOG-provoking tasks. These results are encouraging and enable future work investigating automated FOG assessment during everyday life.

## Background

Parkinson’s disease (PD) is a neurodegenerative disorder that affects over six million people worldwide [1]. One of the most debilitating symptoms associated with PD is freezing of gait (FOG), which develops in approximately 70% of PD patients over the course of their disease [2, 3]. Clinically, FOG is defined as a “brief, episodic absence or marked reduction of forward progression of the feet despite the intention to walk” and is often divided into three manifestations based on leg movement: (1) trembling: tremulous oscillations in the legs of 8–13 Hz; (2) shuffling: very short steps with poor clearance of the feet; and (3) complete akinesia: no visible movement in the lower limbs [1, 4]. While one patient can experience different FOG manifestations, the distribution of manifestations can vary widely among individuals, in which trembling and shuffling are more common than akinetic freezing [5]. The unpredictable nature of FOG poses a significant risk of falls and injuries for PD patients [6, 7, 8], and it can also affect their mental health and self-esteem, leading to a lower quality of life [9]. To relieve the symptoms, dopaminergic medication such as Levodopa is mainly used [10]. During Off-medication states, FOG more commonly occurs [11], while in contrast, FOG episodes are milder in On-medication states but may manifest differently with more trembling [12].

To qualitatively assess FOG severity in PD patients and guide appropriate treatment, subjective questionnaires, such as the Freezing of Gait Questionnaire (FOGQ) and the New Freezing of Gait Questionnaire (NFOGQ), are commonly used [13, 14]. Although these questionnaires may be sufficient to identify the presence of FOG, they are insufficient to objectively describe patients’ FOG severity, and capture treatment effects, as they suffer from recall bias [15], in which the patients may not have been completely aware of their freezing severity, frequency, or impact on daily life. These questionnaires are also poor for intervention studies due to the large test-retest variability resulting in extremely minimal detectable change values [15]. To objectively assess FOG severity, PD patients are asked to perform brief and standardized FOG-provoking tasks in clinical centers. Common tasks include timed-up-and-go (TUG) [16], 180 or 360 degrees turning while walking [17], and 360-degree turning-in-place (360Turn) [18]. The TUG is commonly used in clinical practice since the task includes typical everyday motor tasks such as standing, walking, turning, and sitting. In combination with a cognitive dual-task, it has proven to provoke FOG reliably [19]. Recently, the 360Turn with a cognitive dual-task was also shown to be practical and reliable to provoke FOG for investigating therapeutic effects on FOG [20]. Adding a cognitive dual-task to both the TUG and 360Turn test can increase the cognitive load on individuals, which can result in more FOG events, making these tests more sensitive and perhaps relevant measures of FOG severity in real-life situation [17, 19, 20].

The current gold standard to assess FOG severity during the standardized FOG-provoking tasks is via a post-hoc visual analysis of video footage [17, 21, 22]. This protocol requires experts to label FOG episodes and the corresponding FOG manifestations frame by frame [22]. Based on the frame-by-frame annotations, semi-objective FOG severity outcomes can be computed, such as the number of FOG episodes (#FOG) and the percentage time spent frozen (%TF), defined as the cumulative duration of all FOG episodes divided by the total duration of the walking task [23]. However, this procedure relies on time-consuming and labor-intensive manual annotation by trained clinical experts. Moreover, the inter-rater agreement between experts was not always strong [23], and the annotated #FOG between raters could also contain significant differences due to multiple short FOG episodes being inconsistently pooled into longer episodes [20].

As a result, there is an interest in automated and objective approaches to assess FOG  [5, 24–27]. Traditionally, automatic approaches detect FOG segments based on a predefined threshold for high-frequency spectra of the leg acceleration [28]. These techniques, however, are not fully designed explicitly for FOG as they also provide a positive value to PD patients without FOG and even healthy controls [29]. Additionally, since these techniques rely on rapid leg movements, they may not detect episodes of akinetic FOG. As gait in PD is highly variable, there is increasing interest in deep learning (DL) techniques to model FOG [24, 27, 30–32]. Owing to their large parametric space, DL techniques can infer relevant features directly from the raw input data. As such, our group recently developed a new DL based algorithm using marker-based 3D motion capture (MoCap) data [27]. However, marker-based MoCap is cumbersome to set up and is constrained to lab environments. As a result, inertial measurement units (IMU), due to the better portability, were often used to capture motion signals both in a lab and at home [33, 34] and were widely used for the traditional sensor-based assessment of FOG [24, 31, 35, 36]. The multi-stage temporal convolutional neural network (MS-TCN) stands as one of the current state-of-the-art DL models, initially designed for frame-by-frame sequence mapping in computer vision tasks  [37]. The MS-TCN architecture initially generates an initial prediction using multiple temporal convolution layers and subsequently refines this prediction over multiple stages. In a recent study, a multi-stage graph convolutional neural network was developed specifically for 3D MoCap-based FOG detection. This research demonstrated that the refinement stages within the model effectively mitigate over-segmentation errors encountered in FOG detection tasks  [27]. These errors manifest as long FOG episodes being predicted as multiple short FOG episodes, impacting FOG detection performance of DL models. Acknowledging the necessity of mitigating such errors, approaches like the post-processing step employed in [24] also smooth and merged short FOG episodes in the predicted FOG annotations generated by DL models. Consequently, implementing a post-processing step in FOG annotation from DL models emerges as an essential aspect.

Previous studies proposed automatic FOG detection models for FOG assessment in clinical settings by training and evaluating DL models on datasets that include multiple standardized FOG-provoking tasks measured during both On- and Off-medication states [24, 27, 31, 38]. However, seeing the widespread clinical use of the 360Turn task for FOG detection, it is still uninvestigated if DL models can adequately detect FOG in this task, which forms the first research gap. Additionally, whether training task-specific and medication-specific models enables a better FOG detection performance than a model trained on multiple tasks and both medication states was not discussed in the literature, which forms the second gap.

Moreover, gait patterns and FOG severity can vary substantially among different FOG-provoking tasks [39] and medication states [40, 41]. Prior studies have delved into the impact of medication states on FOG. For instance, researchers in [42] trained a model using a combined dataset of Off and On medication trials and then assessed the model’s performance on each medication state independently. This evaluation aimed to understand how the automatic detection of FOG outcomes derived from the model would respond to medication conditions known to influence FOG severity. Similarly, in [43], investigations were made to determine whether dopaminergic therapy affected the system’s ability to detect FOG. However, these studies have yet to explore the performance of DL models in detecting FOG in an unseen medication state compared to a model trained specifically on data collected from these medication states, which forms the third research gap. Here, “unseen” refers to conditions not included in the model’s training, such as training a model for 360Turn and evaluating its performance on TUG, or training exclusively on On medication data and testing on Off medication data. This gap is critical in evaluating the generalizability of DL models, probing whether their learned features can be robustly applied to new and unseen conditions, ultimately addressing the model’s adaptability beyond its original training context.

Additionally, although these standardized FOG-provoking tasks include walking and turning movements, similar to movements in real-life conditions, they do not include sudden volitional stops, which frequently occur during daily activities at home. Hence, it becomes crucial to be able to distinguish between FOG and volitional stops when transitioning toward at-home FOG assessment. These volitional stops usually do not include any lower limb movements and are often considered challenging to distinguish from akinetic freezing [44]. Although a previous study proposed using physiological signals, such as electrocardiography, to detect discriminative features for classifying FOG from voluntary stops [45], methods using motor signals to distinguish FOG from stops were seldom investigated. To the best of our knowledge, only limited studies proposed FOG detection or prediction on trials with stops using IMU signals [31, 46]. However, while these studies developed models to detect FOG from data that contains voluntary stopping, they did not address the effect of including or excluding stopping instances during the model training phase on FOG detection performance, forming the fourth research gap.

To address the aforementioned gaps, this paper first introduced a FOG detection model to enable automatic FOG assessment on two standardized FOG-provoking tasks (i.e. the TUG task and the 360Turn task) based on IMUs. The model comprises an initial prediction block to generate preliminary FOG annotations and a subsequent prediction refinement block, designed to mitigate over-segmentation errors. Next, we evaluated whether a DL model trained for a specific task (TUG or 360Turn) or a specific medication state (Off or On) could better detect FOG than a DL model trained on all data. In essence, our aim was to ascertain whether DL models necessitate training on task-specific or medication state-specific data. Subsequently, we evaluated the FOG detection performance of DL models when applied to tasks or medication states that were not included during the model training phase. This analysis aims to assess the generalizability of DL models across unseen tasks or medication states. Finally, we investigated the effect of including or excluding stopping periods on detecting FOG by introducing self-generated and researcher-imposed stopping during standardized FOG-provoking tests. Both self-generated and researcher-imposed stops are hereinafter simply referred to as “stopping”. To this end, the contribution of the present manuscript is fourfold:

1. We proposed a FOG detection model for fine-grained FOG detection on IMU data, demonstrating its ability to effectively generalize across two distinct tasks and accommodate both medication states.
2. We show, for the first time, that FOG can be automatically assessed during the 360Turn task.
3. We show that the DL model cannot generalize to an unseen FOG-provoking task, thereby highlighting the importance of expressive training data in the development of FOG assessment models.
4. We show that the DL model can assess FOG severity with a strong agreement with experts across FOG-provoking tasks and medication states, even in the presence of stopping.

The study primarily focuses on evaluating the performance of a state-of-the-art model under different conditions, including different tasks, medication states, and stopping conditions, rather than introducing a novel FOG detection model. A comparison of various FOG detection models is provided in Appendix.

## Methods

### Dataset

We recruited 12 PD patients in this study. Subjects were included if they subjectively reported on the NFOGQ having at least one FOG episode per day with a minimum duration of 5 s. The inclusion criterion was chosen to maximize the chance of capturing FOG in the lab-based assessment procedure. All subjects completed the Montreal Cognitive Assessment (MoCA) [47], Unified Parkinson’s Disease Rating Scale (UPDRS) [48], and Hoehn & Yahr (H&Y) Scale [49] for clinical assessments.

All subjects performed TUG with 180 degrees turning to both directions and a 1-min alternating 360Turn test during the assessments. In the TUG, participants were instructed to stand up from a chair, walk towards a mark placed 2.5 ms from the chair, turn around the mark, walk back to the chair, and sit down. In the 360Turn, participants had to perform rapid alternating 360-degree turns in place for 1 min [20]. While measuring the standardized FOG-provoking tasks, we included a dual task to provoke more FOG episodes [19, 20]. The dual task consisted of the auditory Stroop task [20, 50], in which the words “high” and “low” were played from a computer with both a high and low pitch voice. Participants were instructed to name the pitch they heard and not repeat the word. As a result, the TUGs and 360Turn tests were grouped into one block (two TUG trials and one 360Turn trial). Each block of tests was measured with and without a dual task (6 trials). We also included measurements containing a self-generated or researcher-imposed stopping period to collect data for further training. Each block also consisted of stopping trials, in which TUGs were performed four times, twice with a stop in the straight walking part and twice with a stop in the turning part of the TUG; while 360Turn was performed one time. The block was repeated with self-generated and researcher-imposed stopping (10 trials). All pre-mentioned assessments were done first in the clinical Off-medication state (approximately 12 h after the last PD medication intake) and repeated in the same order during the On-medication state (at least 1 h after medication intake), resulting in 32 trials for each subject. The blocks at each session were performed in randomized order to counter potential fatiguing or motor learning to more or fewer FOGs in the last tests.

All participants were equipped with five Shimmer3 IMU sensors attached to the pelvis and both sides of the tibia and talus. All IMUs recorded at a sampling frequency of 64 Hz during the measurements. RGB videos were captured with an Azure Kinect camera at 30 frames per second for offline FOG annotation purposes. For synchronization purposes, triggered signals were sent at regular intervals of 1 s from the camera to an extra IMU that was connected with a cable to the laptop and synced with the other five IMUs. FOG events were visually annotated at a frame-based resolution by a clinical expert, after which all FOG events were verified by another clinical expert using Elan annotation software [22]. Annotators used the definition of FOG as a brief episode with the inability to produce effective steps [1]. Specifically, a FOG episode started only when the foot of the participant is suddenly no longer producing an effective step forward and is displaying FOG-related features [22]. The episode ended only when it is followed by at least two effective steps (these two steps are not part of the episode) [22]. Unlike previous studies that considered shuffling as one of the FOG manifestations [1, 5], this study adopts a stricter definition of FOG that distinguishes non-paroxysmal shuffling and festination as non-FOG events, although they are probably related to FOG due to the presence of increased cadence with small steps during walking. During model training and testing, these FOG-related events were considered non-FOG events.

### FOG detection model architecture

**Fig. 1:**
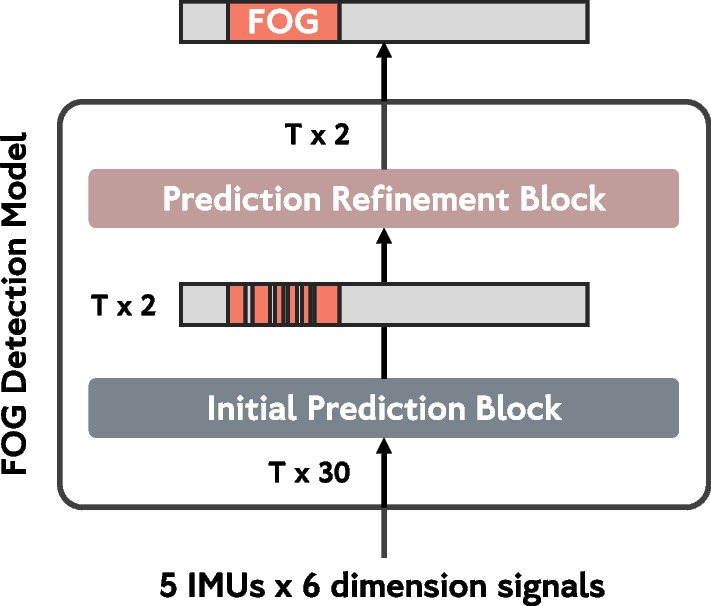
Overview of the proposed FOG detection model architecture. Our proposed FOG detection model comprises two essential blocks: an initial prediction block and a prediction refinement block. The initial prediction block takes the six-dimensional signal of *T* samples from each of the five IMUs and generates initial predictions with the probabilities of positive (FOG) and negative (non-FOG) classifications for each sample within the input sequence. Consequently, the output sequence is structured as $T \times 2$ representing the probabilities of the two classes. The prediction refinement block aims to refine the initial predictions. This block takes the initially predicted probabilities of the two classes as input and applies a smoothing process, removing over-segmentations and enhancing the overall prediction quality. The output of this refinement block is a refined prediction, also structured as $T \times 2$ representing the probabilities of the two classes

The FOG detection model presented in this study consists of two components, as depicted in Fig. 1: (1) an initial prediction block responsible for generating FOG annotations from IMU signals, and (2) a prediction refinement block focused on reducing over-segmentation errors. We conducted comparisons among five FOG detection models for the initial prediction block. Two DL models, namely Long Short Term Memory (LSTM) [51] and Temporal Convolutional Neural Network (TCN) [52], along with three traditional machine learning models, i.e., Support Vector Machine, K Nearest Neighbor, and eXtreme Gradient Boosting (XGBoost), were evaluated. The DL models were trained using raw IMU signals of all five IMUs as input data, while the ML models were trained on 65 features [32] generated from the IMU signals of the talus IMU of both lower limbs. Ultimately, the TCN model outperformed others and was chosen as the initial prediction block. The model comparison results are available in Appendix Table 9.

Similarly, we compared a pre-defined post-processing method [24] with a trained DL model [37] for prediction refinement. The pre-defined post-processing method aimed to merge FOG episodes that were 21 samples apart into a single FOG episode and relabel FOG episodes shorter than 21 samples as non-FOG episodes. The selection of 21 samples was based on the observation that 95% of the FOG episodes in our dataset lasted longer than 0.33 s (21 samples). The trained DL model outperformed the pre-defined post-processing method in performance. Consequently, the trained DL model from [37] was chosen for prediction refinement. A comprehensive comparison between the pre-defined and learned refinement models, as well as the comparison between the inclusion and exclusion of a refinement model, is available in Appendix Table 10.

Based on the conclusion drawn from the comparison presented in Appendix, our proposed FOG detection model employs the TCN from [52] as the initial prediction block and the multi-stage TCN from [37] as the prediction refinement block. A comprehensive visualization of the detailed model architecture is provided in Appendix Fig. 6. Furthermore, specific hyperparameter settings for the two blocks can be found in Appendix Table 11.

### Evaluation

To evaluate the performance of the model, datasets were partitioned using a leave-one-subject-out (LOSO) cross-validation approach. The LOSO cross-validation approach iteratively splits the data according to the number of subjects in the dataset. One subject is evaluated, while the others are used to train the model. This procedure was repeated until all subjects had been used for evaluation. This approach mirrored the clinically relevant scenario of FOG assessment in newly recruited subjects [53], where the model assesses FOG in unseen subjects. The result for all models shown in this study were averaged over all unseen subjects using the LOSO cross-validation approach.

#### Experimental settings

*Clinical setting* To support FOG assessment in clinical settings, which typically do not include stopping, this study first investigated the overall and relative performance of a generic model trained across standardized FOG-provoking tasks that do not include stopping. Next, we assessed generalization across FOG-provoking tasks and medication states by studying the effect of including or excluding training data from a specific task or medication state on detecting FOG.

*Towards the home setting* To move towards FOG assessment in daily life where stopping frequently occurs, we trained and evaluated the performance of a generic model trained across trials with stopping. Next, we assessed the effect of including or excluding stopping periods on detecting FOG.

*Naming convention* The naming convention of all the DL models that were evaluated in this study with their corresponding training data is shown in Table 1. The generic model trained for clinical measurements (i.e., excluding stopping) was termed “Model_Clinical”. Models trained with less data variety were termed (i.e., trained for a specific task or medication state): “Model_TUG”, “Model_360Turn”, “Model_Off”, and “Model_On”. The generic model trained to work towards FOG assessment in daily life (i.e., including stopping) was termed “Model_Stop”. To compare the effect of stopping, we evaluated Model_Clinical and Model_Stop. In order to maintain a similar amount of FOG duration in the training data, Model_Stop was only trained on trials that included stopping.

**Table 1:** Naming convention of the deep learning models evaluated in this study with their corresponding training data

Usage	Model name	FOG-provoking task		Medication state		Stopping	
		TUG	360Turn	Off	On	Exclude	Include
FOG detection in clinical practice	Model_TUG	v		v	v	v	
	Model_360Turn		v	v	v	v	
	Model_Off	v	v	v		v	
	Model_On	v	v		v	v	
	Model_Clinical	v	v	v	v	v	
Towards FOG detection in daily life	Model_Stop	v	v	v	v		v

Models trained for specific tasks or medication states for standardized measurements were termed “Model_TUG”, “Model_360Turn”, “Model_Off”, and “Model_On”. A DL model trained for FOG assessment in the clinical centers, trained on standardized tasks excluding stops, was termed “Model_Clinical”. A DL model trained to work towards FOG assessment in daily life, trained on standardized tasks including stops, was termed “Model_Stop”

#### Metrics

From a clinical perspective, FOG severity is typically assessed in terms of percentage time-frozen (%TF), and the number of detected FOG episodes (#FOG) [23]. This paper used %TF as the primary outcome and #FOG as a secondary outcome based on previous studies [20, 24]. To assess the agreement between the model predictions and the expert annotations for each of the two clinical metrics, we calculated the intra-class correlation coefficient (ICC) with a two-way random effects analysis (random trials, random raters) (ICC (2,1)), in which both the raters and the subjects are treated as random effects, meaning that they are assumed to be a random sample from a larger population [54]. The ICCs between the model and experts were calculated subject-based, with one %TF and #FOG per subject. In other words, the %TF and #FOG were calculated over all trials for each subject. The strength of the agreement was classified according to [55]: $\ge \, 0.80$: strong, 0.6–0.79: moderately strong, 0.3–0.59: fair, and < 0.3: poor.

From a technical perspective, the sample-wise F1 score (Sample-F1) is a metric commonly used in classification problems to evaluate the quality of a model’s predictions at the individual sample level. It provides a balanced measure of a model’s ability to identify positive and negative classes, especially in FOG detection scenarios where the proportion of FOG samples is lower than that of non-FOG samples. When contrasted with metrics such as accuracy, specificity, and sensitivity, the F1 score emerges as a more balanced measure for comparing models’ performances [56]. In binary classification, Sample-F1 is computed by comparing the predicted and true labels. Each sample is classified as true positive (TP), false positive (FP), or false negative (FN) by a sample-wise comparison between the experts’ annotation and model predictions. Sample-F1 is calculated under the formula:

$$F1 = \frac{TP}{TP + \frac{1}{2} (FP + FN)}$$

**Fig. 2:**
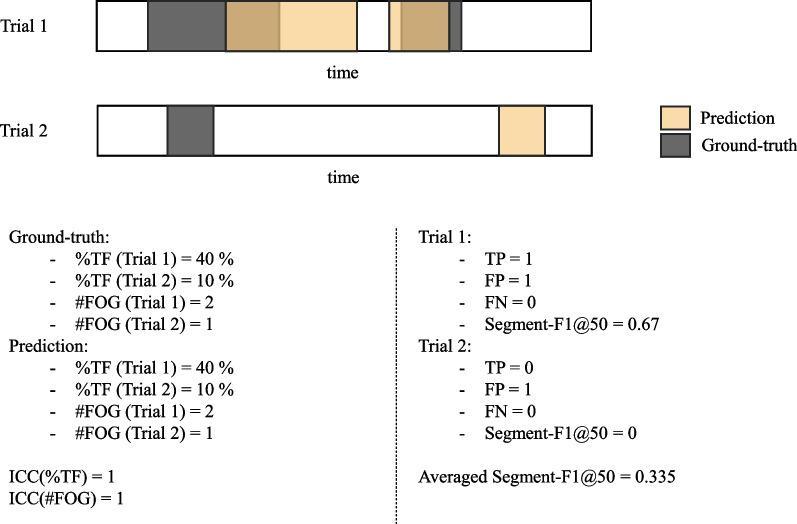
An example for comparing ICC and segment-wise F1 score. This toy example shows the annotations on two trials with the ground-truth annotation as gray and the predicted annotation as yellow. The x-axis represents the timeline for the annotations. When calculating the agreement between the ground-truth and prediction, the %TF and #FOG are both 40% and 2 for the first trial and 10% and 1 for the second trial, resulting in an ICC value of 1. On the other hand, for the segment-wise F1@50 of the first trial, since FP = 1 (the first FOG segment has an IoU less than 50%), TP = 1 (the second FOG segment has an IoU over 50%), and FN = 0, resulting in a segment-F1@50 with 0.67. For the second trial, FP = 1, TP = 0, and FN = 0 resulted in a segment-F1@50 with 0. Thus, the mean Segment-F1@50 equals 0.335. This example shows the disadvantage of using the ICC value of %TF and #FOG to measure the alignment between two annotations

Additionally, the segment-wise F1-score at *k* (Segment-F1@*k*) proposed by Lea et al. [57] is a metric that penalizes over and under-segmentation errors. It allows only minor temporal shifts for the predicted segment, resulting in a much stricter evaluation metric than sample-wise metrics such as Sample-F1 [27]. To compute Segment-F1@*k*, action segments are classified as TP, FP, or FN by comparing the intersection over union (IoU) to a pre-defined threshold *k*. The IoU is calculated as the intersection length of the predicted segment and the ground-truth segment divided by the union between the two segments. If the corresponding IoU of a predicted segment is large than *k*, the predicted segment is TP; otherwise, it is FP. All unpaired ground-truth segments are considered FN. Based on previous studies [27, 58], we set the threshold *k* for IoU as 50% (Segment-F1@50). Additionally, an example to compare %TF, #FOG, and Segment-F1@50 is shown in Fig. 2. The %TF and #FOG for both annotations are 40% and 2 for trial 1, 10% and 1 for trial 2, resulting in a high ICC value of 1. However, the Segment-F1@50 is 0.67 for trial 1 and 0 for trial 2, resulting in an averaged Segment-F1@50 of 0.335. This example shows that although ICC is widely used in previous studies when comparing the inter-rater agreement of %TF and #FOG, it contains the disadvantages of not penalizing shifted annotations, a problem that Segment-F1@50 overcomes. This study calculated one Sample-F1 and Segment-F1@50 for each subject by taking the averaged Sample-F1 and averaged Segment-F1@50 over all trials of that subject. The overall Sample-F1 and Segment-F1@50 under the LOSO cross-validation approach were calculated by averaging the metrics over all subjects.

Based on the above discussion, when comparing the performance between different models, i.e., Model_TUG vs. Model_360Turn, Model_Off vs. Model_On, and Model_Clinical and _Model_Stop, only Sample-F1 and Segment-F1@50 were used. Whereas when showing the agreement between the two generic models and the experts in terms of FOG severity outcomes, the ICC values for %TF and #FOG were reported.

#### Statistical analysis

The Bland-Altman plot [59] was applied to investigate the systematic bias of the %TF and #FOG between the prediction of Model_Clinical and the experts’ annotation. To investigate whether the difference in Sample-F1 and Segment-F1@50 for each subject between two DL models, i.e., Model_TUG vs. Model_360Turn, Model_On vs. Model_Off, and Model_Clinical vs. Model_Stop, was statistically significant, the paired Student’s t-test [60] was applied, with the number of pairs equal to the number of subjects evaluated with LOSO. The homogeneity of variances was verified in all metrics across subjects with Levene’s tests [61]. The Shapiro-Wilk test [62] was used to determine whether the variables were normally distributed across subjects. The significance level for all tests was set at 0.05. All analyses were performed using SciPy 1.7.11, bioinfokit 2.1.0, statsmodels 0.13.2, and pingouin 0.3.12, written in Python version 3.7.11.

## Results

This section first describes the dataset characteristics. Next, we discuss the result of automatic FOG assessments at two levels: (1) FOG detection for clinical measurements with a discussion on the generalization of the FOG detection model and the effect of FOG-provoking tasks and medication states, and (2) FOG detection for moving towards daily life with a discussion on the effect of stopping.

### Dataset characteristics

Table 2 shows the clinical characteristics of the twelve PD patients. Participants varied in their age and disease duration. According to Table 3, a total of 346 trials were collected. Freezing occurred in 38.43% of trials (133 out of 346 trials), with average %TF of 14.62% and total #FOG of 530 observed. The dataset’s mean duration of FOG episodes was 3.01 s, with the shortest episode lasting 0.05 s and the longest episode lasting 63.62 s. Based on the dataset measurement protocol, 32 trials were collected for each subject. Subjects with more than 32 trials was due to repeated measurements, and subjects with less than 32 trials was due to technical difficulties.

**Table 2:** Subject characteristics

	Average ± SD
Age	69.33 ± 6.02
PD duration	12.33 ± 5.99

	Median [Quartile 1 to 3]
MoCA [47]	26.5 [24.25, 26.5, 28]
MDS-UPDRS total [48]	93 [65, 93, 108]
H&Y I/II/III/IV [49]	0/6/3/2

Overview of the age, PD duration, and questionnaire results. The MoCA and MDS-UPDRS total were reported with the median and quartile. The H&Y was reported with the actual numbers in each category, while the data for S3 was missing. All characteristics were measured during the On-phase of the medication cycle

**Table 3:** Dataset characteristics

Subject	Total duration (min)	#Trials	#FOG-trials	#FOG		%TF	FOG episode duration (s)		
							Mean	Min	Max
S1	17.11	29	16	35		19.56	5.73	0.73	32.89
S2	13.90	29	9	34		12.64	3.10	0.05	15.53
S3	13.22	31	6	37		7.11	1.52	0.27	5.20
S4	10.48	27	12	30		7.98	1.67	0.23	7.27
S5	6.88	16	4	22		9.8	1.84	0.31	7.31
S6	12.84	32	1	1		0.1	0.73	0.73	0.73
S7	17.65	32	22	106		14.10	1.41	0.23	12.30
S8	13.48	33	0	0		0.00	N.A.	N.A.	N.A.
S9	14.52	31	15	61		4.49	0.64	0.22	2.62
S10	25.59	21	21	111		52.86	7.31	0.22	63.62
S11	15.62	31	17	74		12.28	1.55	0.09	6.47
S12	20.41	34	10	19		2.07	1.34	0.36	3.64
Sum	181.70	346	133	530	Average	14.62	3.01	0.05	63.62

Overview of the data collected for each subject, including the total duration in minutes, the number of IMU trials (#Trials), the number of FOG trials (#FOG-trials), the percentage of time frozen (%TF), the number of FOG episodes (#FOG), and the mean, min, and max duration of the FOG episodes

The 346 trials in the dataset included 133 trials (81.11 min) collected within the clinical setting, i.e., trials without stopping, and 213 trials (100.60 min) with stopping included. According to Table 4, all 133 trials without stopping were used to train Model_Clinical, while all 213 trials with stopping were used to train Model_Stop. Within the 133 trials without stopping, 89 TUG trials (35.99 min) were used to train Model_TUG, and 44 360Turn trials (45.11 min) were used to train Model_360Turn. Similarly, 67 Off-medication trials (45.75 min) were used to train Model_Off, and 66 On-medication trials (35.36 min) were used to train Model_On. These models were evaluated and discussed in the following sections.

**Table 4:** Overview of the number of trials, total duration (minutes), and FOG outcome of the training data for all models evaluated in this study

Model name	#Trials	Total duration (min)	%TF	#FOG
Model_TUG	89	35.99	19.88	103
Model_360Turn	44	45.11	21.71	179
Model_Off	67	45.75	27.37	205
Model_On	66	35.36	12.53	77
Model_Clinical	133	81.11	20.90	282
Model_Stop	213	100.60	9.57	248

A total of 133 trials were measured with standardized FOG-provoking tests excluding stops, which were used to train Model_Clinical, and 213 trials were measured with self-generated and researcher-imposed stops, which were used to train Model_Stop. Within the 133 trials, 89 trials of TUG tasks were used to train Model_TUG, and 44 were used to train Model_360Turn. Similarly, 67 trials Off medication were used to train Model_Off, and 66 trials were used to train Model_On. Trial durations are shown in minutes. FOG severity is quantified by means of the %TF and #FOG

### Clinical setting: FOG detection

**Fig. 3:**
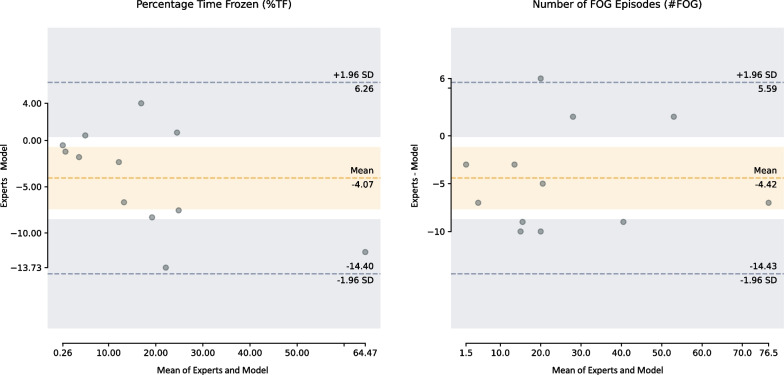
Bland–Altman plot for the clinical metrics from Model_Clinical and the experts. The dots represent the difference in scores per patient on the y-axis (i.e., model’s %TF or #FOG subtracted from experts’ %TF or #FOG), plotted against the mean score per patient from the model and the experts on the x-axis. The orange shaded area represents the 95% CI for the mean bias, and the gray shaded area represents the 95% CI for the upper and lower limits of agreement. A negative mean error indicates that the model overestimates with %TF and #FOG compared with the experts’ annotation

This study first trained and evaluated the proposed model trained for FOG detection in standardized clinical setting (i.e., trials without stopping). The #FOG that Model_Clinical detected per subject varied from 3 to 80, amounting to 335 FOG episodes, while the %TF varied from 0.52 to 70.49%. When comparing with experts’ annotations, the model had a strong agreement in terms of %TF, (ICC = 0.92, CI = [0.68, 0.98]), and #FOG (ICC = 0.95, CI = [0.72, 0.99]). The Bland–Altman plots shown in Fig. 3 revealed a systematic error across FOG severity from the model, with a mean bias of $-4.06$ (CI = [$-7.41, -0.72$]) for %TF and $-4.41$ (CI = [$-7.66, -1.17$]) for #FOG. For %TF, the limits of agreement (LOA) fall within the range of $-14.40$% (CI = $-20.19, -8.59$) to 6.26% (CI = [$-0.45, 12.05$]), showing that it was confident that the differences between the model and the experts would lie in the range of $-14.40$% to 6.26%. For #FOG, the LOA fall within the range of $-14.43$ (CI = [$-20.04, -8.80$]) to 5.59 (CI = [$-0.02, 11.21$]), showing that the differences between the model and the experts will lie in the range of $-14.43$ to 5.59.

**Fig. 4:**
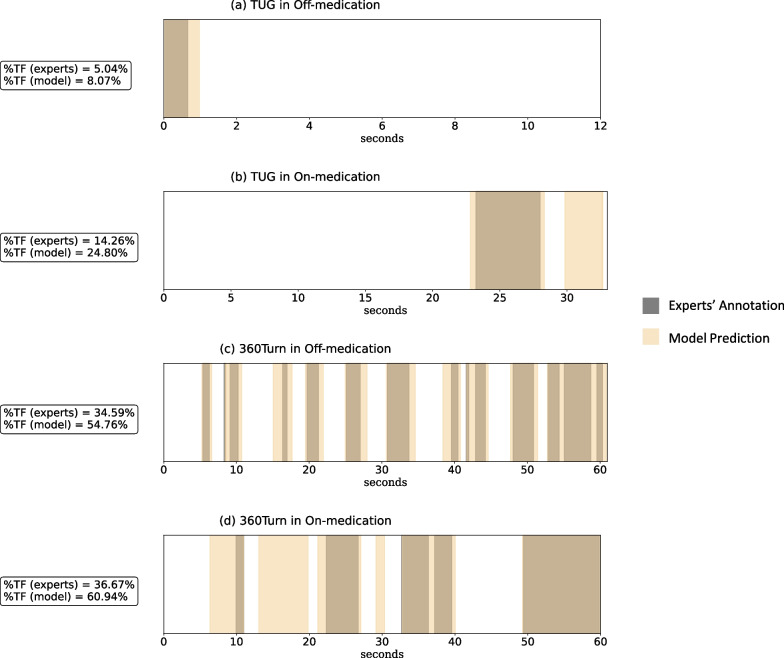
Overview of the annotations for four typical IMU trials from two patients. Four typical trials include annotations for IMU trials measured during four settings: **a** TUG in Off-medication (S3), **b** TUG in On-medication (S1), **c** 360Turn in Off-medication (S3), **d** 360Turn in On-medication (S1). The figures visualize the difference between the manual FOG segmentation by the clinician and the automated FOG segmentation by the DL model. The x-axis denotes the time of the trial in seconds. The gray region indicates the experts’ annotated FOG, and the yellow region indicates the model-annotated FOG. The color gradient visualizes the overlap or discrepancy between the model and experts’ annotations. The figure shows that the model generally annotated broader FOG events compared to experts’ annotation, resulting in a systematic error in %TF shown in Fig. 3

Additionally, when evaluating all standardized trials (i.e., without stopping) within the dataset, results showed that 56.70% of the FP samples were annotated as FOG-related segments, i.e., shuffling and festination, meaning that the model tended to annotate FOG-related samples as FOG. According to the qualitative example of the model and experts’ annotations in Fig. 4, the model generally predicted broader FOG segments compared to the experts’ annotations, resulting in a seeming overestimation of %TF. Also, the model tends to split some experts’ annotated FOG segments into two different FOG segments, resulting in seemingly overestimating #FOG.

Next, we assessed the relative performance of the generic model in detecting FOG for trials with a specific FOG-provoking task, medication state, or with and without stopping. As shown in Table 5, Model_Clinical had a strong agreement with the experts in terms of %TF (all ICCs > 0.92) and #FOG (all ICCs > 0.84). Results showed that it was more difficult for the model to detect FOG in 360Turn tests than TUG in terms of the average Segment-F1@50 (360Turn: 0.45; TUG: 0.67) and Sample-F1 (360Turn: 0.58; TUG: 0.72). Similarly, it was more difficult for the model to detect FOG in Off trials than On trials (Segment-F1@50: 0.55 vs. 0.64; Sample-F1: 0.65 vs. 0.69). However, these results were not reflected in the ICC values for %TF and #FOG, which also shows the inadequate ability of such metrics when comparing different models.

**Table 5:** Overview of the performance of Model_Clinical and Model_Stop

Model	Test data	ICC (%TF)	ICC (#FOG)	Segment-F1@50	Sample-F1
Model clinical	TUG	0.95, CI = [0.86, 0.99]	0.97, CI = [0.89, 0.99]	0.67	0.72
	360Turn	0.94, CI = [0.78, 0.98]	0.84, CI = [0.57, 0.95]	0.45	0.58
	Off-state	0.94, CI = [0.74, 0.99]	0.94, CI = [0.76, 0.99]	0.55	0.65
	On-state	0.92, CI = [0.76, 0.98]	0.90, CI = [0.70, 0.97]	0.64	0.69
	All trials	0.92, CI = [0.68, 0.98]	0.95, CI = [0.72, 0.99]	0.60	0.67
Model stop	TUG	0.78, CI = [0.42, 0.93]	0.77, CI = [0.41, 0.93]	0.65	0.67
	360Turn	0.98, CI = [0.90, 1.00]	0.65, CI = [0.16, 0.89]	0.33	0.47
	Off-state	0.89, CI = [0.52, 0.97]	0.83, CI = [0.53, 0.95]	0.55	0.62
	On-state	0.98, CI = [0.96, 1.00]	0.69, CI = [0.22, 0.91]	0.62	0.64
	All trials	0.95, CI = [0.73, 0.99]	0.79, CI = [0.46, 0.94]	0.59	0.63

We investigated the overall performance of the generic model trained for clinical settings (i.e., excluding stopping) with standardized measurements: Model_Clinical and the generic model trained to work towards FOG detection in daily life (i.e., including stopping): Model_Stop. Results show that Model_Clinical has a strong agreement with the experts in terms of %TF (ICC = 0.92) and #FOG (ICC = 0.95). Also, Model_Stop has a strong agreement with the experts in terms of %TF (ICC = 0.95) and a moderately strong agreement in terms of #FOG (ICC = 0.79). We also showed the relative performance of the two models for each of the four conditions with less data variety: (1) TUG trials, (2) 360Turn trials, (3) Off-medication trials, and (4) On-medication trials. For the four conditions, Model_Clinical was evaluated on trials that excluded stopping, while Model_Stop was evaluated on trials that included stopping

#### Generalization of the FOG detection model

We proceeded to evaluate how Model_Clinical performed in comparison to models specifically designed for various FOG-provoking tasks and medication conditions, including Model_TUG, Model_360Turn, Model_Off, and Model_On. As shown in Table 6, when testing on TUG trials, there was no difference between Model_Clinical and Model_TUG in terms of Segment-F1@50 and Sample-F1. When testing on 360Turn trials, there was no difference between Model_Clinical and Model_360Turn in terms of Segment-F1@50 and Sample-F1. Similarly, When testing on Off-medication trials, there was no difference between Model_Clinical and Model_Off in terms of Segment-F1@50 and Sample-F1. When testing on On-medication trials, there was no difference between Model_Clinical and Model_On in terms of Segment-F1@50 and Sample-F1. While no significant differences emerged between Model_Clinical and the models specifically trained for distinct conditions, tasks, and medication statuses, it’s worth noting that the task-specific models exhibited higher F1 scores compared to the model trained on data with more variability.

**Table 6:** Model comparison results in terms of training on different FOG-provoking task or medication state

Model	Test data	Segment-F1@50		Sample-F1	
		Mean	t-test (#pair)	Mean	t-test (#pair)
Model_TUG	TUG	0.70	t(12) = 0.38, p = 0.710	0.75	t(12) = 0.52, p = 0.612
Model_Clinical	TUG	0.67		0.72	
Model_360Turn	360Turn	0.53	t(12) = 1.24, p = 0.237	0.62	t(12) = 0.86, p = 0.403
Model_Clinical	360Turn	0.45		0.58	
Model_Off	Off	0.52	t(12) = 0.02, p = 0.986	0.66	t(12) = 0.02, p = 0.984
Model_Clinical	Off	0.55		0.65	
Model_On	On	0.68	t(11) = 0.32, p = 0.755	0.76	t(11) = 0.39, p = 0.703
Model_Clinical	On	0.64		0.69	

We investigated the performance of Model_Clinical trained on the two tasks and both medication states with task-specific and medication-specific models. The third and fourth column depicts Segment-F1@50 averaged over all subjects and the paired t-test result. The fifth and sixth columns depict the Sample-F1 averaged over all subjects and the paired t-test result. The number of subjects (pairs) was 12 for TUG, 360Turn, and Off-state, while only 11 subjects were considered for On-state due to technical problems for subject 5 during On-medication state measurements

#### Effect of FOG-provoking tasks and medication states

Next, we investigated the effect of including or excluding data from specific tasks or medication states. As shown in Table 7, when testing on TUG trials, Model_TUG resulted in a statistically higher Segment-F1@50 (p < 0.005) and Sample-F1 (p < 0.005) than Model_360Turn. Similarly, when testing on 360Turn trials, Model_360Turn resulted in a higher Segment-F1@50 and Sample-F1 than Model_TUG, though the differences were not statistically significant. On the other hand, when testing on Off-medication trials, no difference was found between Model_Off and Model_On in terms of Segment-F1@50 (p = 0.952) and Sample-F1 (p = 0.957). Similarly, when testing on On-medication trials, no difference was found between Model_Off and Model_On in terms of Segment-F1@50 (p = 0.579) and Sample-F1 (p = 0.307). The results showed that DL models trained only on TUG trials could still detect FOG in 360Turn trials, while DL models trained only on 360Turn could not detect FOG in TUG trials. In contrast, DL models trained without trials for specific medication states could detect FOG on trials measured during unseen medication states. In other words, the data variance between different FOG-provoking tasks was more challenging to model than between different medication states.

**Table 7:** Model comparison results in terms of training on different FOG-provoking task or medication state

Model	Test data	Segment-F1@50		Sample-F1	
		Mean	t-test (#pair)	Mean	t-test (#pair)
Model_TUG	TUG	0.70	t(12) = 6.19, p < 0.005	0.75	t(12) = 6.07, p < 0.005
Model_360Turn	TUG	0.10		0.15	
Model_360Turn	360Turn	0.53	t(12) = 2.14, p = 0.055	0.62	t(12) = 6.07, p < 0.005
Model_TUG	360Turn	0.44		0.56	
Model_Off	Off	0.52	t(12) = − 0.06, p = 0.952	0.66	t(12) = 0.05, p = 0.957
Model_On	Off	0.53		0.65	
Model_On	On	0.68	t(11) = 0.57, p = 0.579	0.76	t(11) = 1.07, p = 0.307
Model_Off	On	0.65		0.71	

We investigated the generalization of task-specific models to unseen tasks and the generalization of medication-specific models to unseen medication states. The third and fourth column depicts Segment-F1@50 averaged over all subjects and the paired t-test result. The fifth and sixth columns depict the Sample-F1 averaged over all subjects and the paired t-test result. The number of subjects (pairs) was 12 for TUG, 360Turn, and Off-state, while only 11 subjects were considered for On-state due to technical problems for subject 5 during On-medication state measurements

### Towards the home setting: FOG detection with stopping versus clinical ratings

To move towards FOG detection in daily life, we trained and evaluated the DL model, Model_Stop, on trials collected with stopping. As shown in Table 5, when comparing with experts’ annotations, Model_Stop had a strong agreement in terms of %TF, (ICC = 0.95, CI = [0.73, 0.99]), and a moderately stong agreement in terms of #FOG (ICC = 0.79, CI = [0.46, 0.94]). Similar to FOG detection in clinical settings, results show that it was also more difficult for the model to detect FOG in 360Turn tests than TUG in terms of the average Segment-F1@50 (360Turn: 0.33; TUG: 0.65) and Sample-F1 (360Turn: 0.47; TUG: 0.67). Also, it was more difficult for the model to detect FOG in Off trials than On trials (Segment-F1@50: 0.55 vs. 0.62; Sample-F1: 0.62 vs. 0.64).

#### Effect of stopping periods versus no stopping periods

Next, we investigated the effect of stopping periods on FOG detection by comparing the performance of DL models trained on trials with and without self-generated and researcher-imposed stopping, i.e., Model_Clinical and Model_Stop. According to the results shown in Table 8, when evaluating trials collected during standardized measurements, i.e., trials without stopping, there was no difference found between Model_Clinical and Model_Stop in terms of Segment-F1@50 (p = 0.550) and Sample-F1 (p = 0.326).

When evaluating trials collected with stopping periods, the Segment-F1@50 for Model_Stop (mean = 0.60) was significantly higher than Model_Clinical (mean = 0.39; p = 0.005). Similarly, the Sample-F1@50 for Model_Stop (mean = 0.65) was significantly higher than Model_Clinical (mean = 0.44; p < 0.005). Additionally, among the 210 observed stops within the dataset, only 16 (7.61%) were mislabeled as FOG from Model_Stop, while 74 (35.23%) were annotated as FOG from Model_Clinical. The results indicated that the model trained with trials that include stopping could learn to differentiate stopping from FOG, resulting in a statistically higher Segment-F1@50 and Sample-F1 than the model trained without stopping.

**Table 8:** Model comparison results in terms of training with/without trials containing stops

Model	Test data	Segment-F1@50		Sample-F1		
		Mean	t-test (#pair)	Mean	t-test (#pair)	#FP-Stop
Model_Clinical	Non-stop	0.60	t(12) = 0.16, p = 0.874	0.67	t(12) = 0.02, p = 0.979	N.A.
Model_Stop	Non-stop	0.59		0.67		N.A.
Model_Clinical	Stopping	0.40	t(12) =− 5.31, p < 0.005	0.46	t(12) = − 4.39, p < 0.005	74/210
Model_Stop	Stopping	0.59		0.63		16/210

We investigated the effect of including or excluding stopping periods in FOG detection by comparing models trained with (i.e., Model_Stop) and without stopping trials (i.e., Model_Clinical). The third and fourth column depicts Segment-F1@50 averaged over all 12 subjects and the paired t-test result. The fifth and sixth columns depict the Sample-F1 averaged over all 12 subjects and the paired t-test result. The seventh column depicts the number of stops detected as FOG with respect to the total number of stops (#FP-Stop). N.A. was shown for #FP-Stop when testing on trials without stopping as it would not be possible to detect stopping as FOG

## Discussion

This is the first study to show that a DL model using only five lower limb IMUs can automatically annotate FOG episodes frame by frame that matches how clinical experts annotate videos. Additionally, this study is the first to assess the FOG detection performance of a DL model during the dual-task 360Turn task, recently proposed as one of the most effective FOG-provoking tasks [20]. Two clinical measures were computed to evaluate the FOG severity predicted by the DL model trained for the clinical setting (Model_Clinical), the %TF and #FOG [23]. Model_Clinical showed a strong agreement with the experts’ observations for %TF (ICC = 0.92) and #FOG (ICC = 0.95). In previous studies, the ICC between independent raters on the TUG task was reported to be 0.87 [63] and 0.73 [23] for %TF and 0.63 [23] for #FOG, while for 360Turn, the ICC between raters was reported to be 0.99 for %TF and 0.86 for #FOG [20]. While the ICC value in previous studies varied depending on the specific tool and population being studied [64], in comparison, our proposed model achieved similar levels of agreement. This holds significant promise for future AI-assisted FOG annotation work, whereby the DL model annotates FOG episodes initially, and the clinical expert verifies/adjusts only where required. Despite the high agreement with the experts, results showed that the model statistically overestimated FOG severity with a higher %TF and #FOG than the experts when evaluating all trials. The overestimation of %TF and #FOG was partly due to FP when predicting FOG-related movement, such as shuffling and festination, as FOG segments. The systematic overestimation resulted in relatively low F1 scores while maintaining a high ICC. Given that these FOG-related movements often lie on the boundary between freezing and non-freezing [45], it can be challenging for the model to accurately annotate and categorize them in a manner consistent with nearby FOG episodes.

This study aimed to assess the generalization capabilities of DL models across various tasks and medication states by comparing models trained on all tasks and medication states (referred to as Model_Clinical) against task-specific and medication-specific models (Model_TUG, Model_360Turn, Model_Off, and Model_On). Our results showed that task- and medication-specific models performed better than the general model, though these effects were not statistically significant. Moreover, when comparing the performance of the general model on different tasks and medication states, our result showed that it was more difficult for the model to detect FOG in 360Turn tests than TUG in terms of the average Segment-F1@50 and Sample-F1. Also, our result showed that it was more difficult for the model to detect FOG in Off-medication tests than in On-medication tests. Despite evaluating Model_Clinical on both tasks and medication states, our model exhibited relatively lower F1 scores compared to those reported in FOG detection literature [32, 51, 65]. This discrepancy in our study’s F1 scores can be attributed to the challenging nature of our dataset, notably containing a higher proportion of short FOG episodes, with 41.84% lasting less than 1 s. In comparison, the CuPiD dataset [66] has a proportion of 5.06%, while the dataset from [24] reports 0% of such short episodes. When comparing our FOG detection models with those proposed in the literature, detailed in Appendix, we observed that these models struggled to properly detect FOG in our dataset, exhibiting lower Sample F1 scores compared to our model. This disparity suggests that our dataset poses greater difficulty for annotations, possibly due to the prevalence of numerous short episodes.

Our next evaluation focused on determining the extent to which a DL model trained exclusively on a single FOG-provoking task or medication state could generalize to unseen FOG-provoking tasks or medication states. Results showed that the model trained on one FOG-provoking task (i.e., TUG or 360Turn) could better detect FOG in such a task than the model without training on such tasks. Additionally, although previous studies have shown that gait patterns are altered post anti-Parkinsonian medication [40, 41], our results also showed that the model trained on one medication state could still detect FOG in the other medication state. As a result, we recommend caution when applying DL-based FOG assessment models on FOG-provoking tasks that were not explicitly trained on, while applying models trained on different medication states does not show such discrepancies. This also has implications for future work toward daily-life FOG detection. Training data needs to be diversified for all activities encountered during daily. On the other hand, diversifying training data towards the medication states is unnecessary, making data collection more feasible as data can be collected in the On-medication regimens in the future.

While existing approaches utilized DL models to detect FOG on standardized FOG-provoking tasks with IMUs [24, 31], the model’s ability to distinguish FOG from stopping remains undetermined, which is critical for free-living assessment [45]. Therefore, voluntary and instructed stops were introduced in the standardized FOG-provoking tasks. When evaluating trials without stops, results showed no difference between the model trained without stops and the model trained with stops, showing that adding stopping periods in the training data does not affect the DL model to detect FOG. Additionally, when evaluating trials with stops, results showed that compared with the model trained without stops, the model trained with stops produced less FP of stopping (16 compared to 74). While it was considered that stops are difficult to distinguish from FOG with movement-related signals, especially for akinetic FOG [67], our model could still detect FOG in the presence of stops. Moreover, our result highlights the importance of including stopping in the training data.

Although this study has provided valuable insights, there are some limitations to acknowledge. The first limitation is that the videos in our dataset were annotated sequentially by two clinical experts. The first annotator’s work was verified and, if needed, corrected by the second annotator. As a result, we could not calculate the inter-rater agreement in our study to compare our models’ annotation against. However, the literature shows that inter-rater agreement is 0.39–0.99 [20, 23, 24, 27, 35, 63] and that these differences between experts were sometimes due to minor differences between FOG and FOG-related movements. Our DL model’s agreement with the clinical experts exceeded those previously published inter-rater agreements, and just as between experts, most of our model’s mispredicted FOG segments were marked as FOG-related segments by the experts. Future work could investigate the development of DL models that can better differentiate between FOG and FOG-related events. On the other hand, whether such differentiation is truly needed depends on the research or clinical question. The second limitation is that this study simulated free-living situations by asking patients to stop when performing standardized FOG-provoking tasks. Yet, free-living movement will contain substantially more variance (e.g., daily activities) than captured during our standardized tasks. Moreover, FOG severity during our tasks does not necessarily represent FOG severity in daily life [44, 68]. Therefore, future work should establish the reliability of our approach to data measured in free-living situations. The third limitation is that this study showed that training DL models with trials that include stopping resulted in better performance in detecting FOG in trials that include stopping. However, whether DL models are able to distinguish between FOG and stopping for all manifestations of FOG (e.g., akinetic FOG) remains to be investigated. The fourth limitation is our choice of utilizing the complete sensor configuration, which includes all five IMUs in this study. Previous research has compared various IMU positions and recommended an optimal technical setup comprising only three IMUs (specifically, lumbar and both ankles) [24]. We included the performance results of models trained with the 3-IMU configuration in Appendix. The result demonstrate that there is no significant difference between the performance of models trained with five IMUs and three IMUs. However, additional research is required to definitively establish the ideal sensor configuration for effective FOG detection in home environments. The fifth limitation is the small number of participants compared to the other use cases in DL literature. As this study evaluated the model with the LOSO cross-validation approach, the results still showed that the model could generalize learned features to unseen subjects. Moreover, despite the small number of subjects, the number of samples and FOG events in the dataset used in this study is comparable with the literature [27, 31]. Future studies could evaluate automatic FOG assessment on larger datasets or across datasets. The sixth limitation is that the recruited PD patients subjectively reported having at least one FOG episode per day with a minimum duration of 5 s. While the proposed model works for these severe freezers, it still has to be verified whether the model also generalizes to mild freezers.

## Conclusion

This paper introduced a DL model comprising an initial prediction block and a prediction refinement block for IMU-based FOG assessment trained across two FOG-provoking tasks in both On- and Off-medication states and trials containing stopping. We established that the proposed DL model resulted in strong agreement with experts’ annotations on the percentage of time frozen and the number of FOG episodes. This highlights that a single DL model can be trained to generalize over FOG-provoking tasks and medication states for FOG assessment in a clinical setting. Additionally, our investigation revealed that while there was no significant difference observed between the model trained on all-encompassing data and task- and medication-specific models. Moreover, we established that DL models should include specific FOG-provoking tasks in the training data in order to be able to detect FOG in such a task, while this is not necessary for different medication states. Finally, we established that the proposed model can still detect FOG in trials that contain stopping. Though, only when stopping is included in the training data. These findings are encouraging and enable future work to investigate FOG assessment during everyday life.

## Figures and Tables

**Fig. 5:**
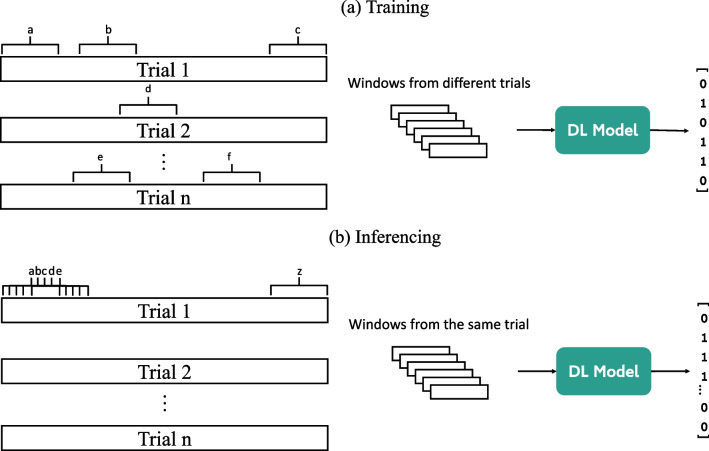
An example for comparing window generation during model training and inferencing. This example illustrates the utilization of windows extracted from various IMU trials in both model training and inference stages. During model training, windows generated from different trials were randomly chosen to train the DL model. The actual label assigned to each window corresponded to the experts’ annotation of the middle sample within that window. During the inference phase, windows were generated from the same IMU trial, employing a step size of 1 sample. Subsequently, all generated windows were input into the DL model to conduct sample-wise predictions

**Fig. 6:**
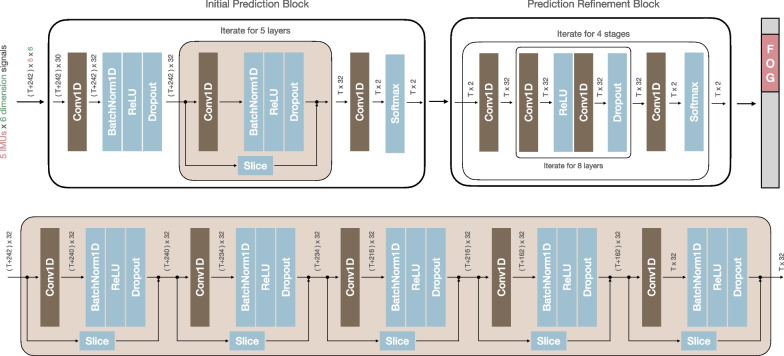
Detailed model architecture of the FOG detection model. Our proposed FOG detection model comprises two essential blocks: an initial prediction block and a prediction refinement block. The initial prediction block utilizes the TCN proposed by Pavllo et al. [52], featuring five temporal convolution layers with valid convolutions. This TCN transforms the input sequence (padded with 121 samples on both sides) of shape $(T+242)\times 30$ into an output of shape $T\times 2$. The prediction refinement block, leveraging a multi-stage TCN architecture proposed by Farha and Gall [37], aims to refine the initial predictions. The multi-stage TCN comprises four stages of ResNet-style TCN, each containing eight temporal convolution layers with the same convolutions. The output of this refinement block is a refined prediction, also structured as $T\times 2$, representing the probabilities of the two classes

## Data Availability

The input set was imported and labeled using Python version .2.7.12 with Biomechanical Toolkit (BTK) version 0.3 [71]. The model architecture was implemented in Pytorch version 1.2 [72] by adopting the public code repositories of MS-TCN [37], and VideoPose3D [52]. All models were trained on an NVIDIA GeForce RTX 2080 GPU using Python version 3.7.11. The datasets analyzed during the current study are not publicly available due to restrictions on sharing subject health information.

## Appendix

### FOG detection model design

IMU-based FOG detection models typically adopt window-based methodologies, dividing an IMU trial into predefined windows to train models for FOG detection. Given a window, FOG detection models aims to classify the window into non-FOG or FOG. The need for predicting FOG annotations frame by frame, similar to experts’ annotations, demands a sliding mechanism with a one-sample step size during evaluation. However, this sliding operation sometimes leads to over-segmentation of FOG annotations  [27, 58]. To mitigate these errors, researchers have proposed post-processing methods  [24]. These methods aim to refine initial annotations and eliminate short FOG episodes that are shorter than the smallest FOG episode in the dataset. On the other hand, employing a refinement model  [27, 58] presents a more flexible approach, bypassing the need for extensive knowledge about dataset characteristics.

We propose a FOG detection model comprising an initial prediction block to generate initial FOG annotations and a prediction refinement block to smooth and refine these initial predictions. We initially compare five FOG detection models for the initial prediction block and then compare two approaches for refining the initial predictions.

#### Problem definition

An IMU trial can be represented as $X \in \mathbb {R}^{T \times C_{in}}$, where *T* is the number of samples and $C_{in}$ is the input feature dimension ($C_{in} = 30$ for 5 IMUs with 3 dimensional acceleration and gyroscope). Each IMU trial *X* is associated with a ground truth label vector $Y \in \mathbb {R}^{T \times L}$, where *L* is the number of output classes, i.e., 2 for non-FOG and FOG. To generate predictions for each sample, the model learns a function $f: X \rightarrow \hat{Y}$ that transforms a given input sequence $X=x_0,\ldots,x_{T-1}$, where $x_i \in \mathbb {R}^{1 \times C_{in}}$, into an output sequence $\hat{Y}=\hat{y}_0,\ldots,\hat{y}_{T-1}$, where $y_i \in \mathbb {R}^{L}$ that closely resembles the manual annotations *Y*.

#### Initial prediction block

Our primary objective is to determine the state-of-the-art method for initial FOG prediction. FOG detection models are typically categorized into two main types: Feature-based models, which extract predefined features from IMU data within the window, and Signal-based models, which directly use raw data for FOG detection. Consequently, we selected two distinct signal-based models extensively employed in FOG detection literature: LSTM [51] and TCN [27]. These models were trained on raw IMU signals. Additionally, we evaluated three established traditional machine learning models commonly utilized for FOG detection as feature-based models: Support Vector Machine (SVM) with a radial basis function kernel, K Nearest Neighbors (KNN), and XGBoost. These models were trained on 65 pre-defined features used for FOG detections, as outlined in [32].

The comparisons were conducted on our dataset with 12 subjects, with the partition that excludes instances of stopping. This partition aimed to assess the FOG detection models specifically for clinical detection purposes. For both model training and testing, each IMU trial underwent segmentation into windows of length *Q*, generated with a step size of 1 sample. Every window was assigned a ground truth label represented by the middle sample of the ground truth annotation within that particular window. All window pairs generated from the dataset were utilized in training the models. During the inference phase, we segmented each trial into *T* fixed-length sequences, each sequence having a length of *Q*. Subsequently, these sequences were processed by the model to generate *T* predictions for each trial across the two classes *L*. In inference scenarios, the predicted output is formalized as a 2D matrix $\hat{Y} \in \mathbb {R}^{T \times L}$. An example illustrates the utilization of windows extracted from different IMU trials in both model training and inference stages is shown in Fig. 5.

#### Neural network model design: signal-based models

For the DL models, we adopted typical architectures documented in the literature. The LSTM network configuration consisted of passing the input sequence through two bidirectional LSTM layers, each comprising 32 cells. This LSTM network transformed the input sequence of shape $Q \times C_{in}$ into an internal representation of shape $Q \times 32$. Subsequently, an average pooling layer was employed for temporal pooling, resulting in an output of shape $1 \times 32$. The output was passed through a linear layer followed by a softmax layer, generating probabilities for the two classes $1 \times L$, where $L=2$.

Regarding the TCN network, we used the architecture from [52]. The TCN architecture has a single TCN block comprising five temporal convolution layers. Employing a kernel size of 3, dimensionality of 32, and dilation rates designed to cover the sequence length *Q*, this TCN utilized valid convolutions, directly transforming the input sequence of shape $Q \times C_{in}$ into an output of shape $1 \times 32$. The output was passed through a linear layer with a softmax activation function, generating probabilities for the two classes. The detailed model architecture, specifically elucidating how valid convolutions are executed within the TCN model, can be found in the original study [52] (Fig. 6).

For both DL models, the experiments utilized the Amsgrad optimizer [69] with a learning rate of 0.0005, decayed with a factor of 0.95 for each epoch. The beta1 and beta2 parameters in Amsgrad were set to 0.9 and 0.999, respectively. For consistency, the window size (*Q*) for both DL models was set to 256, corresponding to a 4-s window. All DL models were trained for 50 epochs. A class-weighted categorical cross-entropy loss function was applied. Before training and testing the models, all six channels of the IMU signals for each trial were centralized by subtracting the mean value of each signal to remove the constant bias.

#### Machine learning model design: feature-based models

To compare the performance of signal-based models with traditional FOG detection models utilizing pre-defined features, we selected three widely used ML models as representatives. All IMU trials underwent segmentation into windows of length *Q* with a step size of 1. Here, *Q* equaled 64, 128, or 256 samples, corresponding to window sizes of 1, 2, and 4 s, respectively. Each IMU window served as the basis for computing 65 features, following the methodology proposed in [32]. These features were derived from IMU data captured on both lower limbs, resulting in a total of 130 features for analysis. It’s important to note that features generated from magnetometers were excluded from our study due to the absence of this sensor modality in our dataset.

The SVM was evaluated for adjusting the cost parameter (0.1, 1, 10, 100, 1000), gamma (1, 0.1, 0.01, 0.001, 0.0001), and window size *Q* (64, 128, 256). In the case of KNN, tuning encompassed variations in the number of neighbors (ranging from 1 to 50), different distance metrics (manhattan distance and euclidean distance), and window size *Q* (64, 128, 256). For XGBoost, the tuning process involved optimizing the max depth (ranging from 2 to 10), number of estimators (ranging from 60 to 220 with a step of 40), learning rate (1, 0.1, 0.01, 0.001, 0.0001), and window size *Q* (64, 128, 256). The reported results only encompass the performance of the ML models that exhibited the best hyperparameter configuration.

#### Evaluation

For evaluating and comparing model performance, we primarily reported the sample F1 score. Notably, the Segment-F1@50 metric was omitted from the comparison of initial prediction models due to extensive over-segmentation observed in the predictions made by these models. Consequently, all models displayed uniformly low Segment-F1@50 scores, rendering comparative analysis ineffective. The evaluation process for all models employed the LOSO cross-validation approach.

To assess the models’ performance based on the sample F1 score, we conducted paired sample t-tests. These tests compared the best model against each of the other models, with the number of pairs equating to the number of subjects evaluated using LOSO. To avoid type-I errors, p-values were adjusted for multiple comparisons, as defined in Li [70]. The significance level for all tests was set at 0.05 to determine statistically significant differences in performance across models.

#### Results

The results showcased in Table 9 demonstrate that the TCN model achieves the highest F1 score. Upon conducting statistical tests, it was observed that the F1 score for the TCN was significantly better than all feature-based ML models. However, no statistically significant difference was found between the TCN and the LSTM model. The hyperparameter settings of the chosen model, namely TCN [52], are detailed in Table 11.

**Table 9:** Initial prediction model comparison results

Model	Sample-F1	
	Mean	t-test
SVM	0.28	p = 0.008
KNN	0.16	p = 0.001
XGBoost	0.27	p = 0.001
LSTM	0.40	p = 0.497
TCN	0.50	N.A.

### Prediction refinement block

Previous studies have revealed that employing ML models for fine-grained FOG detections might result in splitting long freezing episodes into numerous smaller ones [27, 35], consequently causing over-segmentation. Subsequent to identifying the optimal initial prediction model, we proceeded to compare the efficacy of two different approaches aimed at mitigating this over-segmentation issue: (1) A pre-defined smoothing approach outlined in [24], which doesn’t involve training a model, and (2) Utilization of a DL model trained without pre-defined information, as proposed in [27].

For our evaluation process, we chose the TCN model outlined in [52] as the initial prediction model.

#### Pre-defined post-processing method

We implemented the pre-defined post-processing method introduced in [24]. This method involves combining model-identified FOG periods separated by a single example into a singular FOG event. Additionally, short FOG periods lasting only one example were reclassified as non-FOG instances. In our dataset analysis, it was observed that 95% of FOG episodes persisted for durations longer than 0.33 s (21 samples). To retain at least 95% of these FOG episodes after post-processing, the method combined FOG episodes in the initial prediction that were 21 samples apart into a single FOG episode. Concurrently, FOG episodes shorter than 21 samples were relabeled as non-FOG instances.

#### Deep learning refinement method

The DL refinement method employed in this study aimed to train a DL model for prediction refinement. We applied the refinement model derived from the MS-TCN architecture initially proposed in [37]. This model’s design involved the input sequence undergoing processing through four ResNet-style TCN blocks, each consisting of eight layers. These layers employed a kernel size of 3, with a dimensionality of 32 and dilation rates set at factors of 2 (1, 2, 4, 8, 16, 32, 64, 128).

The training of the DL refinement model utilized the Amsgrad optimizer [69] with a learning rate of 0.0005 and decayed with a factor of 0.95 for each epoch. The beta1 and beta2 parameters in Amsgrad were set to 0.9 and 0.999, respectively. The DL models were trained for 50 epochs, employing a combination of class-weighted categorical cross-entropy loss function and smoothing loss [37]. For the smoothing loss, parameters $\tau$ and $\lambda$ were set to 4 and 0.15, respectively.

#### Evaluation

Model performance evaluation was conducted based on reported sample F1 scores and Segment-F1@50. To compare the models, paired sample t-tests were conducted, with the significance level set at 0.05. These tests aimed to assess statistical differences between the performance of the two models based on both F1 scores.

#### Results

The results obtained are summarized in Table 10. Notably, the comparison emphasizes that the trained DL model achieves a statistically higher Segment-F1@50 score compared to the pre-defined post-processing method. While the Sample-F1 score for the trained DL model was also higher than the pre-defined post-processing method, the difference did not reach statistical significance. Moreover, a comparison was conducted between the model predictions with and without the addition of a DL refinement approach as a prediction refinement block. As depicted in Table 10, incorporating a prediction refinement block resulted in statistically higher Segment-F1@50 and Sample-F1 scores. These findings strongly indicate that the strategy of training a refinement model significantly improves the smoothness of the initial prediction. This improvement signifies better generalization compared to relying on a pre-defined post-processing approach. Particularly, the pre-defined approach necessitates knowledge of the shortest FOG episode duration within a dataset to avoid overly smoothing and merging of predicted short episodes. Consequently, based on these findings, this study opted for utilizing the trained DL model for post-processing instead of relying on a pre-defined approach. The hyperparameter settings of the chosen model are detailed in Table 11.

**Table 10:** Prediction refinement method comparison results

Model	Segment-F1@50		Sample-F1	
	Mean	t-test	Mean	t-test
Initial prediction block + Pre-defined approach	0.54	p = 0.020	0.64	p = 0.192
Initial prediction block + DL refinement approach	0.60		0.67	
Initial prediction block	0.32	p < 0.005	0.50	p = 0.008
Initial prediction block + DL refinement approach	0.60		0.67

**Table 11:** Hyperpamareter settings for the selected initial prediction and prediction refinement models

Model	Type	Parameter	
Initial prediction (TCN )	Model architecture	#Hidden features	32
		#TCN layers	5
		Dilation for each TCN layer	1, 3, 9, 27, 81
	Training procedure	#Epochs	50
		Batch size	1024
		Optimizer	Amsgrad (beta = (0.9, 0.999))
		Learning rate	0.0005
		Learning rate decay (per epoch)	0.95
		Loss function	Weighted cross-entropy loss
Prediction refinement (multi-stage TCN )	Model architecture	#Hidden features	32
		#Stages	4
		#TCN layers	8
		Dilation for each TCN layer	1, 2, 4, 8, 16, 32, 64, 128
	Training procedure	#Epochs	50
		Batch size	1
		Optimizer	Amsgrad (beta = (0.9, 0.999))
		Learning rate	0.0005
		Learning rate decay (per epoch)	0.95
		Loss functions	Weighted cross-entropy loss + smoothing loss ($\tau$ = 4, $\lambda$ = 0.15)

### Comparison of models trained with different IMU sensor positions

While a prior study [24] had proposed an optimal technical setup utilizing three IMUs (specifically, lumbar and both ankles) following an extensive comparison of various IMU, we compared our full 5-IMU sensor configuration with the previously recommended best technical 3-IMU setup (lumbar and both ankles) detailed in [24] for FOG detection training on trials without stopping. Our comparative study employed a model that integrated the best-performing initial prediction model, the TCN from [52], along with the refinement model from [37].

As shown in Table 12, the model trained with 5 IMUs has a higher Segment-F1@50 and Sample-F1 compared to the model trained with 3 IMUs. However, no statistically significant differences were observed in terms of both F1 scores.

**Table 12:** Model comparison results in terms of training with different number of IMUs

IMU configuration	Segment-F1@50		Sample-F1	
	Mean	t-test	Mean	t-test
5-IMU	0.60	p = 0.388	0.67	p = 0.850
3-IMU	0.57		0.66	

## Abbreviations

PD: Parkinson’s disease
FOG: Freezing of gait
FOGQ: Freezing of Gait Questionnaire
NFOG-Q: New Freezing of Gait Questionnaire
MoCA: Montreal Cognitive Assessment
UPDRS: Unified Parkinson’s Disease Rating Scale
H&Y: Hoehn & Yahr
ML: Machine learning
DL: Deep learning
TUG: Timed-up-and-go
360Turn: 360-Degree turning-in-place
%TF: Percentage time spent frozen
#FOG: Number of FOG episodes
IMU: Inertial measurement unit
MoCap: Motion-captured
TCN: Temporal convolutional network
MS-TCN: Multi-stage temporal convolutional neural network
LOSO: Leave-one-subject-out
SD: Standard deviation
TP: True positive
TN: True negative
FP: False positive
FN: False negative
ICC: Intra-class correlation coefficient
CI: Confidence interval
BTK: Biomechanical Toolkit
N.A.: Not applicable
